# Congenital Tuberculosis in a Stillborn Calf

**DOI:** 10.3390/pathogens15010114

**Published:** 2026-01-20

**Authors:** María Fiorella Alvarado Pinedo, Adrián Di Paolo, Martín Zumárraga, Federico Illanes, Damián Moyano, Luis María Peralta, Gabriel Travería

**Affiliations:** 1 Centro de Diagnóstico e Investigaciones Veterinarias (CEDIVE), Facultad de Ciencias Veterinarias, Universidad Nacional de La Plata, La Plata 1900, Buenos Aires, Argentina; falvarado@fcv.unlp.edu.ar (M.F.A.P.); ladipaolo@fcv.unlp.edu.ar (A.D.P.); fillanes@fcv.unlp.edu.ar (F.I.); luisperaltavet@yahoo.com.ar (L.M.P.); 2 Instituto de Agrobiotecnología y Biología Molecular, Instituto Nacional de Tecnología Agropecuaria (INTA-CONICET), Hurlingham 1686, Buenos Aires, Argentina; zumarraga.martin@inta.gob.ar (M.Z.); moyanord@gmail.com (D.M.)

**Keywords:** tuberculosis, *Mycobacterium bovis*, congenital, transmission

## Abstract

Bovine tuberculosis is a zoonotic infectious disease of cattle caused by the bacterium *Mycobacterium bovis*. In adult cattle, transmission is mainly via the respiratory route, whereas, in young calves, oral infection is also common. Congenital tuberculosis is rare. The purpose of this study was to describe in utero infection of a bovine stillbirth. The fetus was necropsied and tissue samples were processed for histopathology, bacteriology and PCR; a sample of the isolated strain was genotyped using spoligotyping. The whole herd was tuberculin skin tested and the dam’s serum was also assessed for the presence of antibodies against bovine tuberculosis with indirect ELISA. The macroscopic findings in different organs were consistent with tuberculosis. The histopathology demonstrated typical granulomatous lesions in the liver, spleen, hepatic and mediastinal lymph nodes. Acid-fast bacilli were observed in the smears stained using the Ziehl–Neelsen method. The colonies isolated were PCR-positive for IS6110 and the spoligotype corresponded to SB0140. The dam of the stillborn was positive for indirect ELISA and reacted to a single caudal skin test with bovine tuberculin. The fetal infection in utero with *Mycobacterium bovis* was confirmed through necropsy, histopathology and bacteriology, reinforcing the importance of testing young animals.

## 1. Introduction

Bovine tuberculosis, also known as mammalian tuberculosis, is a chronic disease of cattle caused by a group of closely related mycobacteria members of the *Mycobacterium tuberculosis complex*, mainly *Mycobacterium bovis* [1,2], which also affect a wide range of animal species, including humans [3]. This disease is a zoonosis with a significant economic burden on the cattle industry due to its direct and indirect costs, such as those related to animal testing, slaughtering, replacement, etc. [4]. In Argentina, bovine tuberculosis is currently under mandatory national control (Servicio Nacional de Sanidad y Calidad Agroalimentaria; SENASA). The control is based on testing and culling after the identification of reactor cattle with a single caudal bovine tuberculin test. The rationale of the control relies on the culling of infected animals before infectious individuals can transmit the mycobacterium to another susceptible animal [5]. Due to the chronic condition of this disease, most infected animals are subclinical. This chronic character and the lack of sensitivity in diagnostic tests hinder bovine tuberculosis eradication [6]. The transmission of bovine tuberculosis varies with the age of the animals. In adult cattle, transmission occurs mainly via the respiratory route [7], whereas milking calves are mainly infected via the oral route through the ingestion of contaminated colostrum or milk from a tuberculous cow [8]. Also, wildlife can pose a risk of transmission of bovine tuberculosis, acting as hosts [9,10]. Other forms of transmission are not commonly described. For instance, congenital transmission is exceptional in all species and is seldom reported [11]. Due to some epidemiological data, other ways of transmission are under debate [12]. The present study aims to describe a bovine stillborn with a lesion compatible with tuberculosis and isolation of *Mycobaterium bovis*.

## 2. Materials and Methods

History of the herd: One stillborn calf from a five-year-old cow, belonging to a beef herd consisting of 102 Aberdeen Angus cows with no recorded history of tuberculosis, was subjected to necropsy and gross examination. The circumstances of the stillborn’s dam delivery were not disclosed by the owner of the farm located in the Province of Buenos Aires, Argentina.

Necropsy and sampling: The necropsy was performed according to standard procedures [13]. For histopathological examination, samples of heart, brain, kidney, lung, liver, spleen, mediastinal and liver lymph nodes were cut into 0.5 cm-wide sections and fixed by immersion in 10% neutral buffered formalin for 24 h. The fixed tissues were embedded in paraffin, cut into 4 µm thick sections, and stained with hematoxylin and eosin (H&E).

Bacteriology: Samples of liver, spleen, mediastinal and hepatic lymph nodes were collected under sterile conditions and stained with the conventional Ziehl–Neelsen method [14]. The samples were decontaminated with sodium hydroxide according to Petroff’s method [15], and, after decontamination, 100 μL of the samples was seeded onto Stonebrink and Lowenstein Jensen medium and incubated at 37 °C for 60 days [16].

PCR: The DNA was extracted from colonies isolated in Stonebrink medium, and a loop of scraped bacterial soma was diluted in 200 mL of distilled DNAse-free water, heated in a dry bath for 40 min at 98 °C, and centrifuged at 12,000 rpm for 10 min [17]. The DNA obtained was stored at −20 °C for subsequent analysis. The detection of IS6110, specific to *Mycobacterium tuberculosis* complex, was performed by Touch Down PCR (IS6110 TD-PCR) [18] using primers INS1 (5′cgtgagggcatcgaggtggc3′) and INS2 (5′gcgtaggcgtcggtgacaaa3′), as previously described [19,20], which amplify a 245 base pair DNA fragment. The PCR products were submitted to horizontal electrophoresis on a 2% agarose gel in 1× TAE buffer stained with ethidium bromide and visualized under ultraviolet transilluminator.

Spoligotyping: The spoligotyping of the *Mycobaterium bovis* isolates was performed following the instructions previously published [21]. The PCR products were hybridized on a spoligotyping membrane with oligo-nucleotides of spacer sequences, using a miniblotter according to the manufacturer’s instructions (MapMyGenome). The membrane was incubated with streptavidin–peroxidase and the spacers were detected by ECL chemiluminescence (Pierce ECL Western Blotting Substrate, Thermo Fisher Scientific, Waltham, MA, USA), followed by exposure of an X-ray film to the membrane. The patterns visualized in the X-ray film were compared with those contained in the database on the *Mycobacterium bovis* website of the Complutense University of Madrid, Spain (http://www.mbovis.org). To analyze and visualize the hypothetical relationship of genetic patterns of the strains, we applied an eBURST algorithm by using the PhyloViz free software (https://online.phyloviz.net/index, accessed on 22 December 2025).

Immunological tests: Serum from the dam of the stillborn calf was subjected to in-house indirect ELISA, as previously described [22]. An intradermal tuberculin test was performed on the rest of the herd in accordance with local regulations [23].

## 3. Results

**Figure 1:**
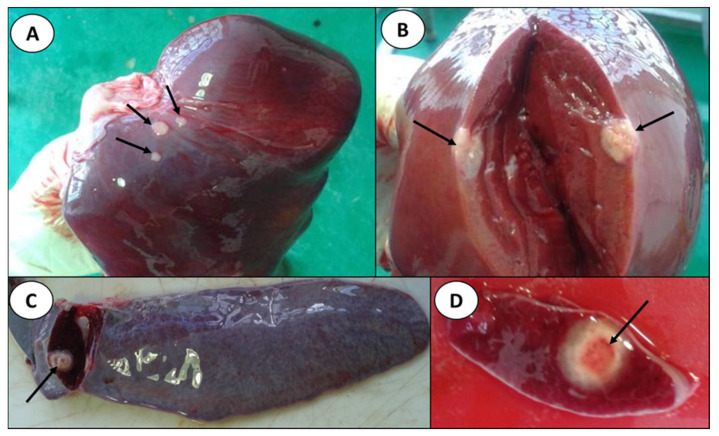
(**A**): Macroscopic visualization of the liver showing three granulomas protruding from the surface. (**B**): Macroscopic visualization of the liver with cut surface showing a granuloma. (**C**): Macroscopic visualization of the spleen showing a granuloma inside the parenchyma. (**D**): Macroscopic visualization of the spleen with a closer approach to the cut surface showing a granuloma with a reddish center. The black arrows point to the granulomas.

Necropsy: The stillborn calf was a male, weighing 11 kg, with mild autolysis, and of gestational age at term. Gross pathology revealed multifocal granulomatous hepatitis; the lesions were mainly located on the diaphragmatic face of the right lobe, below the Glisson’s capsule, generating a slight protrusion of it (Figure 1A). These lesions had a circular shape, with a white surface, and a size that varied between a few millimeters and up to 3 cm in diameter. Sections of the granulomas exhibited a caseous center, and some showed varying degrees of calcification (Figure 1B). The spleen presented focal granulomatous splenitis. This lesion, about 3 cm in diameter, was located on the parietal surface, close to the hilum (Figure 1C,D). The hepatic, mediastinal, cranial sternal and thoracic aortic lymph nodes presented multifocal or focal granulomatous lymphadenitis, with lesions of variable size, with a caseified center and, in some cases, a calcified one.

Bacteriology: The Ziehl–Neelsen staining method revealed a scarce presence of acid-fast bacilli similar to the range scale 1 described by García-Jiménez et al. [24]. After 2 months of incubation in Stonebrink medium, the colonies resembling mycobacteria were subjected to the Ziehl–Neelsen staining method, confirming the presence of acid-fast bacilli, and then subjected to PCR.

Molecular detection: The presence of *Mycobacterium tuberculosis* complex from the culture was confirmed by IS6110 TD-PCR results. The spoligotyping showed the characteristic spoligotype of *Mycobacterium bovis* and was identified as SB0140.

**Figure 2:**
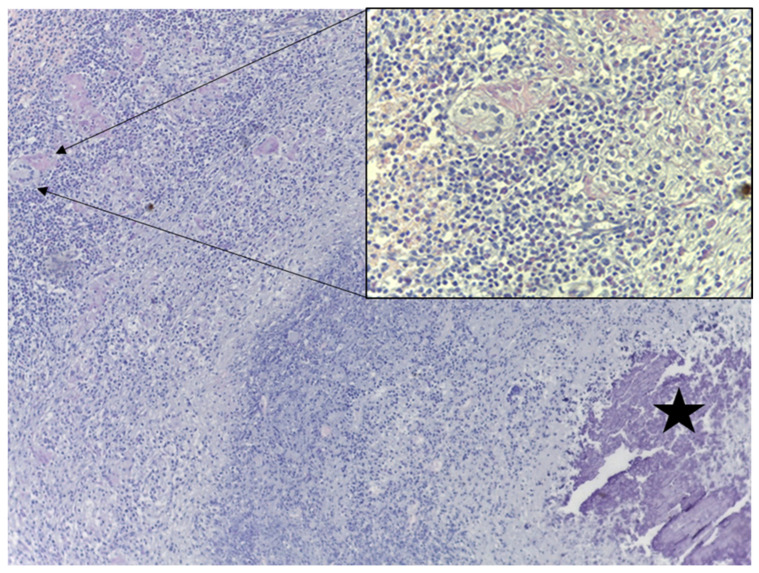
Liver granuloma: the black star at the bottom right side depicts the center of the granuloma with extensive foci of caseous necrosis, with peripheral infiltration of epithelioid cells, lymphocytes and Langhans multinucleated giant cells (H&E 100×). Insert: higher magnification of a Langhans multinucleated giant cell (H&E 450×).

Histopathology: The histopatological examination revealed foci of caseous necrosis with zones of mineralization in the spleen and lymph nodes, with the evident presence of numerous Langhans multinucleated giant cells and epithelioid cells surrounding the necrotic areas. A few occasional foci of epithelioid cells surrounded by lymphocytes were observed in the alveolar parenchyma of the lung. The liver showed extensive foci of caseous necrosis, with peripheral infiltration of lymphocytes and Langhans multinucleated giant cells. Some small inflammatory foci made up of a few epithelioid cells and lymphocytes were also found (Figure 2).

Immunological tests: 5 out of the 102 animals skin tested with tuberculin were positive to tuberculin, including the dam of the stillborn. In addition, two were suspicious, and the dam was also positive for tuberculosis according to indirect ELISA.

## 4. Discussion

The presentation of stillborns with tuberculosis is uncommon. However, a few cases have been described in a 45-day-old calf [25], a 15-day-old calf [26], a 25-day-old calf [27], and a 21-day-old calf [28]. In adult cattle, the primary route of infection is usually the respiratory route, through inhalation of aerosolized mycobacteria in droplets [29,30]. In calves, the oral route is common after ingestion of contaminated milk. If the infection progresses, regardless of the primary route, mycobacteria may reach other organs by hematogenous or lymphatic routes [29]. In some cases, mycobacteria may also reach the female genital tract [31]. The outcomes of infection in utero will depend on the stage of gestation: infection at early stages usually ends in fetal mortalities, whereas infection at later stages causes abortion or perinatal mortalities [32]. In the present study, we describe visible lesions compatible with tuberculous granulomas in the liver and spleen of a stillborn calf, suggesting that the infection occurred approximately after 125 days of gestation, after the development of fetal immune antigen recognition capacity [32]. At necropsy, visible lesions of diverse sizes were localized in the spleen and liver. In a previous study, visible lesions were observed in the abdomen of 2-week-old calves, 20 weeks after challenge with *Mycobacterium bovis* [12]. In humans, previous studies have proposed transplacental transmission via the umbilical vein with the primary complex in the liver, followed by secondary hematogenous spread, as well as through the ingestion or aspiration of infected amniotic fluid with the formation of the primary complex in the gastrointestinal tract or the lungs [26,33].

In this case, the fetal capacity to mount an immune response in the uterus able to develop granulomas similar to those seen in calves and adult cattle is remarkable. This capacity is demonstrated by the microscopic findings that revealed the presence of granulomas with Langhans multinucleated giant cells, epithelioid cells, lymphocytes, and foci of caseous necrosis, in different organs. In some zones, the microscopic findings also showed mineralization classified between stages III and IV based on lesions from adult cattle [34]. Under experimental conditions, these stages were found to occur 35-90 days after infection [35]. In the present study, the isolation of the *Mycobacterium tuberculosis* complex in Stonebrink medium was confirmed by TD-PCR, and genotyping using the spoligotype method allowed the *Mycobacterium bovis* spoligotype SB0140, which is the prevailing spoligotype in Argentina, to be identified [17,36,37]. The dam of the stillborn belongs to a tuberculin-positive herd, identified as positive from the tuberculin test and with detectable antibodies against bovine tuberculosis using ELISA. This is in agreement with previous studies that have correlated humoral responses with increased pathology and disseminated disease [38,39].

To prevent the dissemination of the disease, the confirmation of tuberculosis in this stillborn reinforces the importance of testing at an early age before infected animals transmit the disease to other susceptible animals. The presence of lesions demonstrates the capacity of the fetus to mount delayed-type hypersensitivity at an early age. Previous works have mentioned how tuberculin performance varies with many factors and stresses the importance of testing young animals as good responders to tuberculin with the highest lesion confirmation risk [28,40,41]. In some instances, the failure of the tuberculin test-based eradication programs stems from the presence of anergic animals. The occurrence of this complicated phenomenon increases if an infected animal is not identified as soon as the diseases progresses [42,43].

## Data Availability

Any further information related to the current study is available from the corresponding author.
